# *Pcsk6* Deficiency Promotes Cardiomyocyte Senescence by Modulating Ddit3-Mediated ER Stress

**DOI:** 10.3390/genes13040711

**Published:** 2022-04-18

**Authors:** Wenxing Zhan, Liping Chen, Hongfei Liu, Changkun Long, Jiankun Liu, Shuangjin Ding, Qingyu Wu, Shenghan Chen

**Affiliations:** 1 Vascular Function Laboratory, Human Aging Research Institute, School of Life Science, Jiangxi Key Laboratory of Human Aging, Nanchang University, Nanchang 330031, China; zhanwenxing@email.ncu.edu.cn (W.Z.); 355600210009@email.ncu.edu.cn (L.C.); 352428818018@email.ncu.edu.cn (H.L.); longchangkun@email.ncu.edu.cn (C.L.); 2 Aging and Vascular Diseases, Human Aging Research Institute, School of Life Science, Jiangxi Key Laboratory of Human Aging, Nanchang University, Nanchang 330031, China; liujiankun@email.ncu.edu.cn (J.L.); 402404718072@email.ncu.edu.cn (S.D.); 3 Cyrus Tang Hematology Center, Collaborative Innovation Center of Hematology, State Key Laboratory of Radiation Medicine and Prevention, Soochow University, Suzhou 215123, China; qywu88@yahoo.com

**Keywords:** cardiomyocytes, DDIT3, ER stress, PCSK6, senescence

## Abstract

Cardiac aging is a critical determinant of cardiac dysfunction, which contributes to cardiovascular disease in the elderly. Proprotein convertase subtilisin/kexin 6 (PCSK6) is a proteolytic enzyme important for the maintenance of cardiac function and vascular homeostasis. To date, the involvement of PCSK6 in cardiac aging remains unknown. Here we report that PCSK6 expression decreased in the hearts of aged mice, where high levels cyclin dependent kinase inhibitor 2A (P16) and cyclin dependent kinase inhibitor 1A (P21) (senescence markers) were observed. Moreover, PCSK6 protein expression was significantly reduced in senescent rat embryonic cardiomyocytes (H9c2) induced by D-galactose. *Pcsk6* knockdown in H9c2 cells increased P16 and P21 expression levels and senescence-associated beta-galactosidase activity. *Pcsk6* knockdown also impaired cardiomyocyte function, as indicated by increased advanced glycation end products, reactive oxygen species level, and apoptosis. Overexpression of *PCSK6* blunted the senescence phenotype and cellular dysfunction. Furthermore, RNA sequencing analysis in *Pcsk6*-knockdown H9c2 cells identified the up-regulated DNA-damage inducible transcript 3 (*Ddit3*) gene involved in endoplasmic reticulum (ER) protein processing. Additionally, DDIT3 protein levels were remarkably increased in aged mouse hearts. In the presence of tunicamycin, an ER stress inducer, DDIT3 expression increased in *Pcsk6*-deficient H9c2 cells but reduced in *PCSK6*-overexpressing cells. In conclusion, our findings indicate that PCSK6 modulates cardiomyocyte senescence possibly via DDIT3-mediated ER stress.

## 1. Introduction

Aging is a principal risk factor for cardiovascular disease, the leading cause of mortality worldwide. The prevalence of cardiovascular disease is more than 75% for people 60–79 years old and above 89% for people ≥ 80 years old [1]. The most common age-related cardiovascular diseases include hypertension, coronary heart disease, heart failure, and atrial fibrillation [2]. Cardiac aging is defined as gradual structural changes and functional deterioration due to cellular and molecular alterations associated with increasing age [3]. Cardiomyocytes account for more than two-thirds of the cell mass of the heart. The myocardium degenerative changes contribute to cell loss, mitochondrial dysfunction, abnormal cardiac remodeling, and diastolic dysfunction [4,5,6]. In aging hearts, the cardiomyocyte number is reduced, whereas the proportion of senescent cells is increased [7]. Impaired morphology and function have been reported in cardiomyocytes in aging hearts [7].

Various genes and molecular pathways, including the natriuretic peptide system, have been attributed to cardiomyocyte senescence [8]. Atrial natriuretic peptide (ANP) is a cardiac hormone that is derived from its precursor (pro-ANP) by corin-mediated proteolytic cleavage [9,10]. There is evidence that old rats display decreased pro-ANP levels in the atria and that aging impairs ANP production, contributing to heart failure and hypertension [11]. In addition, ANP variants impact on cardiovascular responses to exercise in advanced ages [12]. At cellular levels, down-regulation of ANP occurred in senescent cardiomyocytes [13].

Our recent study has linked proprotein convertase subtilisin/kexin type 6 (PCSK6, also known as PACE4) to its downstream corin-ANP cascade, which plays a critical role in maintaining cardiac function and normal blood pressure [14,15]. PCSK6 deficiency and corin mutations cause cardiovascular diseases such as heart failure and hypertension [15,16,17]. Several studies demonstrate that PCSK6 may activate transforming growth factor-β (TGF-β) [18,19] and that TGF-β signals transduction changes in the epigenetic state through microRNAs, thereby promoting cellular senescence and heart aging [20]. PCSK6 has also been found to control cardiac and vascular remodeling in acute myocardial infarction and atherosclerosis [21,22]. Other investigations reveal that knockdown of PCSK6 induces cell apoptosis [23,24] and that *Pcsk6* mutant mice develop premature ovarian aging [25]. RNA microarray studies have also shown the down-regulation of *Pcsk6* expression in the hearts of old rats [26]. To date, however, the effect and molecular mechanisms of PCSK6 on cardiac aging have yet to be elucidated.

In this study, we investigated the impact of PCSK6 on cardiac aging and underlying molecular mechanisms using an aged mouse model and senescent cardiomyocytes induced by D-galactose (D-gal). We show that PCSK6 is involved in cardiomyocyte senescence in a mechanism likely mediated by endoplasmic reticulum (ER) stress.

## 2. Materials and Methods

### 2.1. Animal Experiments

C57BL/6 mice were obtained from the Model Animal Research Center of Nanchang University. The mice were maintained in ventilated cages under standard conditions and allowed to take food and water freely. Only male mice were recruited in the study. All animal procedures were carried out following the Guide for the Care and Use of Laboratory Animals of the Human Aging Research Institute of Nanchang University.

### 2.2. Cell Culture and Treatment

H9c2 cells, rat heart–derived embryonic myocytes, were purchased from the American Type Culture Collection (ATCC, Manassas, VA, USA) and cultured in Dulbecco’s Modified Eagle Medium (DMEM, Gibco, Grand Island, NY, USA) with 10% fetal bovine serum (FBS, Hyclone, Logan, UT, USA), 100 U/mL penicillin, and 100 U/mL streptomycin (Sigma-Aldrich, St. Louis, MO, USA) at 37 °C in humidified incubators with 5% CO_2_. When at ~80% confluency, the cells were treated with 10 g/L of D-gal (Sigma-Aldrich, St. Louis, MO, USA) in glucose-free DMEM (Gibco, Grand Island, NY, USA) for 48 h and used for further experiments.

### 2.3. SiRNA and Plasmid DNA Transfection

Cells were transfected with 50 nM siRNA or 0.5 μg/mL plasmid expressing human *PCSK6* [14] using Lipofectamine 2000 (Invitrogen, Carlsbad, CA, USA) in Opti-MEM (Gibco, Grand Island, NY, USA) following the manufacturer’s instructions. siRNAs included a non-targeting control siRNA and two sets of siRNAs (si*Pcsk6*-1 and si*Pcsk6*-2) targeting the rat *Pcsk6* gene (GenePharma, Suzhou, Jiangsu, China). The siRNA sequences (sense strand) were as follows: control siRNA: 5′-UUC UCC GAA CGU GUC ACG U-3′; si*Pcsk6*-1: 5′-GCA CAC AAC UGC UUC UCA A-3′; and si*Pcsk6*-2: 5′-UGA CCG UGA CAG AUC UCA C-3′. The cells were collected at 48 or 72 h after transfection.

### 2.4. RNA Sequencing and Analysis

H9c2 cells were transfected with siRNAs for 72 h. Three replicate samples were collected for each group. Total RNAs were prepared and used for library construction, sequencing, and data analysis on the BGISEQ-500 platform (Beijing Genomics Institute, Shenzhen, Guangdong, China).

### 2.5. Immunofluorescent Staining

Cells or frozen tissue sections were incubated with 4% paraformaldehyde for fixation for 15 min and then treated with permeabilization buffer for 10 min. After blocking with 1% BSA and 10% FBS for 2 h, the samples were incubated with a primary antibody at 4 °C overnight. The antibodies used in the study were those against PCSK6 (1:400, Abcam, Cambridge, UK), advanced glycation end products (AGEs) (1:200, Abcam, Cambridge, UK), and DNA-damage inducible transcript 3 (DDIT3) (1:500, CST, Danvers, MA, USA). The secondary fluorescence-labeled antibodies (1:200, Biolegend, San Diego, CA, USA) were incubated for 1 h in the dark. After washing, nuclei were stained with Hoechst (Beyotime, Shanghai, China). Images were captured by a confocal microscope (Carl Zeiss, Oberkochen, BW, Germany).

### 2.6. Hoechst Staining

Cells were prepared and fixed as mentioned above. To stain nuclei, 5 μg/mL of Hoechst33342 (Beyotime, Shanghai, China) was added and incubated at room temperature for 30 min in the dark. Images were taken using the confocal microscope. Five fields were randomly selected for each sample and the percentage of shrunken nuclei was calculated.

### 2.7. Senescence-Associated β-Galactosidase (SA-β-Gal) Staining

SA-β-gal activity was measured with Senescence β-Galactosidase Staining Kit (Beyotime, Shanghai, China). Briefly, the cells were fixed with formaldehyde at room temperature for 10 min. After washing with PBS, the cells were stained by fresh X-gal solution at 37 °C overnight. The cells were inspected under an optical microscopy, and the percentages of positive cells were calculated.

### 2.8. RNA Isolation and Quantitative Real-Time PCR (qPCR)

Total RNAs from cells were extracted by TRIzol reagent (Invitrogen, Carlsbad, CA, USA) and 1000 ng of RNA was used for reverse-transcription using a kit (Zomanbio, Beijing, China) following the manufacturer’s instructions. qPCR was performed by SYBR Green system (Mei5bio, Beijing, China). Fold changes in gene expression were calculated by the 2^−ΔΔCT^ method. The primer sequences were as follows: rat Pcsk6, 5′-CCA GTC TCG CTC ACG GAT G-3′ and 5′-CGC AGC CTT TAT CAC CAC AC-3′; rat interleukin 6 (Il6), 5′-GTT TCT CTC CGC AAG AGA CTT C-3′ and 5′-TGT GGG TGG TAT CCT CTG TGA-3′; rat Ddit3, 5′-CAC ACC TGA AAG CAG AAA CCG-3′ and 5′-GGA CAC TGT CTC AAA GGC GA-3′; rat Gapdh, 5′-CTC ATG ACC ACA GTC CAT GC-3′ and 5′-TAC ATT GGG GGT AGG AAC AC-3′.

### 2.9. Protein Extraction and Western Blotting

Cells or tissues were lysed in a RIPA buffer (Solarbio, Beijing, China) supplemented with PMSF. After homogenizing for 5 min, the lysates were centrifuged at 13,000× *g* for 30 min. Protein concentration was measured using BCA Protein Assay Kit (Beyotime, Shanghai, China). Proteins in the lysates were separated by SDS-PAGE and transferred to PVDF membranes (Millipore, Billerica, MA, USA). The membranes were blocked with 5% skim milk for 1 h and incubated with primary antibodies including those against PCSK6 (1:4000, Abcam, Cambridge, UK), P16 (1:1000, Abcam, Cambridge, UK), P21 (1:1000, Abcam, Cambridge, UK), DDIT3 (1:1000, CST, Danvers, MA, USA), and GAPDH (1:10,000, Proteintech, Wuhan, Hubei, China) at 4 °C overnight. Horseradish peroxidase-conjugated secondary antibodies were used for visualization (ABclonal, Wuhan, Hubei, China). The protein bands were detected by SuperSignal ECL detection kit (Beyotime, Shanghai, China). Quantification of band intensities, normalized to GAPDH, was carried out using ImageJ (NIH, Bethesda, MD, USA).

### 2.10. Measurement of Reactive Oxygen Species (ROS)

Cells were incubated with 2′,7′-Dichlorofluorescein diacetate (DCHF-DA) (Sigma-Aldrich, St. Louis, MO, USA), a fluorogenic ROS indicator, at 37 °C for 20 min in the dark. Subsequently, the cells were digested with Trypsin solution and examined using flow cytometer (BD Bioscience, Franklin Lakes, NJ, USA), and the data were analyzed by flowjo v10 software (BD Bioscience, Franklin Lakes, NJ, USA).

### 2.11. Measurement of Apoptotic Activity

Apoptotic activity in cells was measured by an Annexin V-FITC apoptosis detection kit (BestBio, Shanghai, China), according to the manufacturer’s instructions. Briefly, the cells were washed with pre-cooled PBS and resuspended in Annexin V binding solution. Next, the cell suspension was incubated with Annexin V-FITC on ice for 15 min in the dark and then with Propidium Iodide for 5 min. The cells were analyzed by flow cytometry (BD Bioscience, Franklin Lakes, NJ, USA).

### 2.12. Statistical Analysis

Data were analyzed using GraphPad Prism 7.0. All values are shown as the mean ± SD. Statistical analysis was performed by two-tailed Student’s *t*-test for two groups and one-way ANOVA followed by Tukey multiple comparison for three or more groups. A *p* value < 0.05 was considered as statistically significant.

## 3. Results

### 3.1. PCSK6 Protein Expression Is Reduced in the Heart of Aged Mice

**Figure 1:**
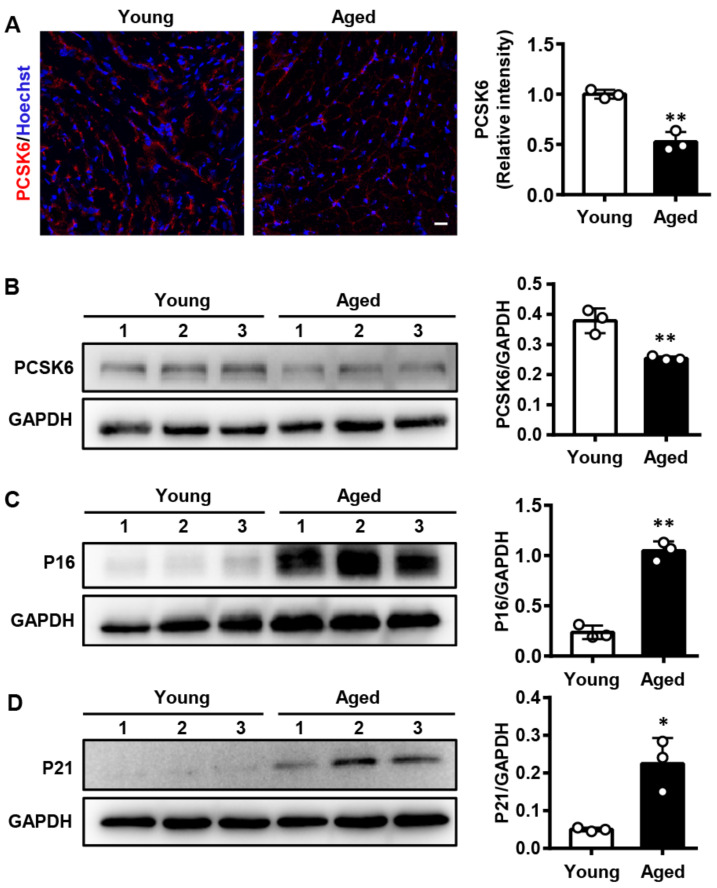
Decreased PCSK6 protein expression in aged mouse hearts. (**A**) Immunofluorescent staining for PCSK6 (red) and nuclei (blue) in frozen heart sections from young mice (3 months, *n* = 3) and aged mice (24 months, *n* = 3). Scale bar: 20 µm. Relative levels were calculated according to mean fluorescent intensity. (**B**–**D**) Western blot analysis of PCSK6, P16, and P21 protein levels in hearts from young mice (3 months, *n* = 3) and aged mice (24 months, *n* = 3). Relative expression, normalized to GAPDH level, was assessed by densitometry. Values are mean ± S.D. * *p* < 0.05, ** *p* < 0.01 vs. Young.

Based on the microarray data of decreased *Pcsk6* mRNA levels in aged rats [26], we examined PCSK6 protein expression in a natural aging mouse model using immunofluorescent staining and Western blotting. We found that PCSK6 protein expression was reduced in hearts of aged mice (24 months) compared to those of young mice (3 months) (Figure 1A,B). Moreover, protein levels of senescence markers P16 and P21 were higher in hearts of aged mice than those of young mice (Figure 1C,D). The results indicate that PCSK6 expression decreases with age.

### 3.2. PCSK6 Expression Is Decreased in the Senescent Cardiomyocytes Induced by D-Gal

**Figure 2:**
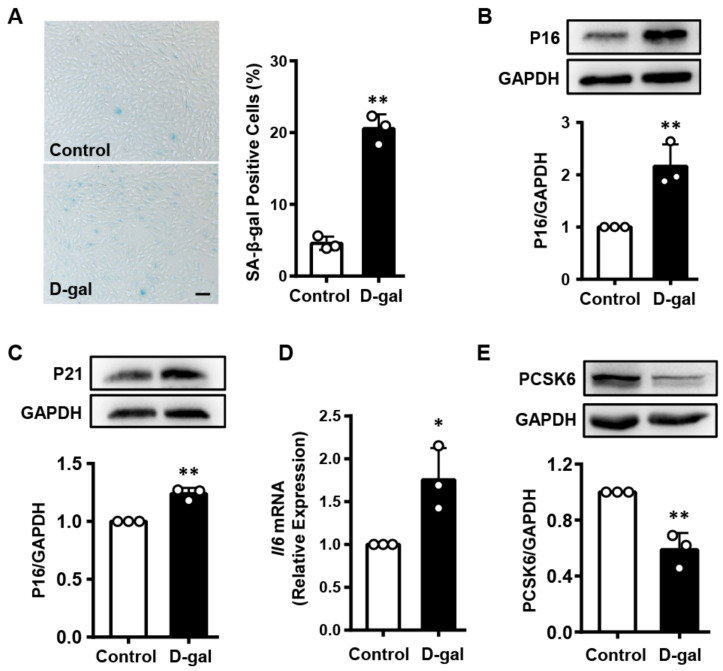
Decreased PCSK6 expression in senescent cardiomyocytes induced by D-gal. (**A**) SA-β-gal staining for H9c2 cells with D-gal treatment. Scale bar: 100 µm. Percentages of blue-stained cells were calculated. (**B**,**C**) P16 and P21 protein levels in D-gal-treated H9c2 cells were estimated by densitometric quantification of Western blots. (**D**) *Il6* mRNA expression in H9c2 cells treated with D-gal by qPCR. (**E**) Western blot analysis of PCSK6 protein expression in H9c2 cells in the presence of D-gal. Values are mean ± S.D. * *p* < 0.05, ** *p* < 0.01 vs. Control.

To verify the above result at the cellular level, we established a model of D-gal-induced cardiomyocyte senescence using H9c2 cells, a model for in vitro study of cardiomyocytes [27]. SA-β-gal activity, a marker for senescent cells, increased, as indicated by high percentages of blue-stained H9c2 cells treated with D-gal, compared to the percentages in control cells (Figure 2A). In Western blotting, P16 and P21 protein levels also increased in D-gal-treated H9c2 cells (Figure 2B,C). In qPCR quantification assay, elevated *Il6* mRNA levels were observed in D-gal-treated H9c2 cells (Figure 2D). These data indicate that D-gal treatment induced cardiomyocyte senescence. By Western blotting, we found that PCSK6 protein expression decreased in the senescent H9c2 cells (Figure 2E). These results suggest that PCSK6 is involved in cardiomyocyte senescence.

### 3.3. Pcsk6 Gene Knockdown Promotes Cardiomyocyte Senescence

**Figure 3:**
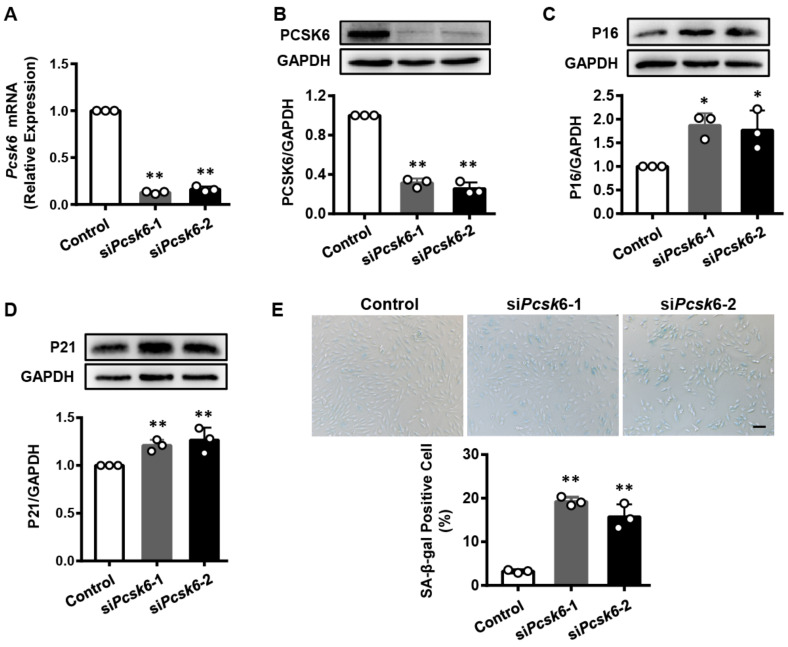
Increased cardiomyocyte senescence by *Pcsk6* knockdown. (**A**) *Pcsk6* mRNA expression in H9c2 cells treated with two sets of siRNAs targeting rat *Pcsk6* gene (si*Pcsk6*-1 and si*Pcsk6*-2) was estimated by qPCR. (**B**) PCSK6 protein expression in si*Pcsk6*-transfected H9c2 cells was analyzed by densitometric quantification of Western blotting. (**C**,**D**) Western blot analysis of P16 and P21 protein levels in si*Pcsk6*-transfected H9c2 cells. (**E**) SA-β-gal staining in si*Pcsk6*-transfected H9c2 cells and quantitative data for SA-β-gal-positive cells. Scale bar: 100 µm. Data are means ± S.D. * *p* < 0.05, ** *p* < 0.01 vs. Control.

We next investigated whether *Pcsk6* deficiency induces cardiomyocyte senescence. In qPCR, we found a decrease in *Pcsk6* mRNA levels in H9c2 cells treated with two sets of siRNAs (si*Pcsk6*-1 and si*Pcsk6*-2) compared to the non-targeting siRNA control (Figure 3A). Likewise, PCSK6 protein levels were reduced in these cells (Figure 3B). Conversely, P16 and P21 expression levels increased in *Pcsk6*-knockdown cells compared with those in the control cells (Figure 3C,D). Consistently, the proportion of SA-β-gal positive cells was also higher in the *Pcsk6*-knockdown cells (Figure 3E). All these findings indicate that cardiomyocytes lacking PCSK6 exhibit cellular senescence.

### 3.4. Pcsk6 Gene Knockdown Impairs Cardiomyocyte Function

**Figure 4:**
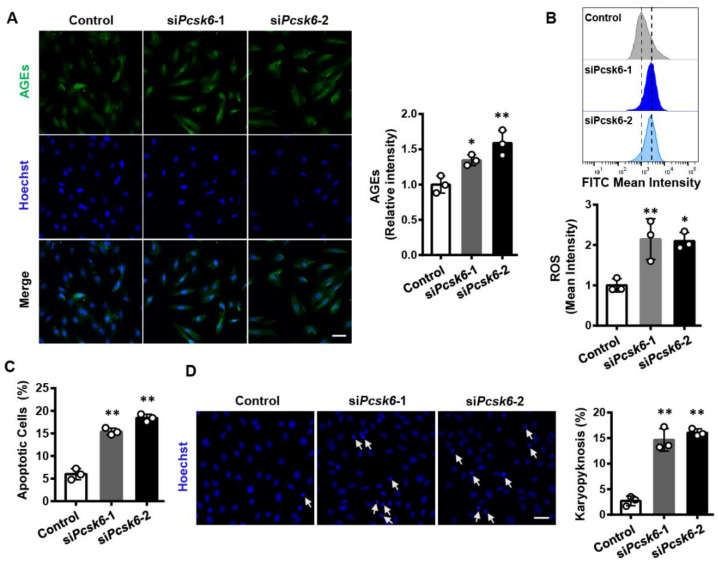
Impaired cardiomyocyte function by *Pcsk6* knockdown. (**A**) Immunofluorescent staining for AGEs (green) and nuclei (blue) in si*Pcsk6*-transfected H9c2 cells. Scale bar: 40 µm. Analysis of quantitative data was conducted based on mean fluorescence intensity. (**B**) ROS production from si*Pcsk6*-transfected H9c2 cells by flow cytometry with the FITC channel. (**C**) Apoptosis in si*Pcsk6-*transfected H9c2 cells detected by Annexin V-FITC in flow cytometry. (**D**) Hoechst 33342 staining for apoptosis in si*Pcsk6*-transfected H9c2 cells. Scale bar: 40 µm. Arrows indicate karyopyknosis. Data are means ± S.D. * *p* < 0.05, ** *p* < 0.01 vs. Control.

To determine whether *Pcsk6* deficiency reduces cardiomyocyte function, we tested AGEs, ROS, and apoptosis in H9c2 cells transfected with two sets of targeting siRNAs. Elevated AGE levels were detected by immunofluorescent staining in *Pcsk6*-knockdown cells (Figure 4A). Similarly, ROS levels increased under these conditions (Figure 4B). *Pcsk6*-knockdown H9c2 cells also showed increased apoptosis (Figure 4C). The result was supported by Hoechst staining (Figure 4D). These data show that cardiomyocyte function is impaired after *Pcsk6* knockdown.

### 3.5. PCSK6 Overexpression Reverses Cardiomyocyte Senescence and Dysfunction Induced by D-gal

**Figure 5:**
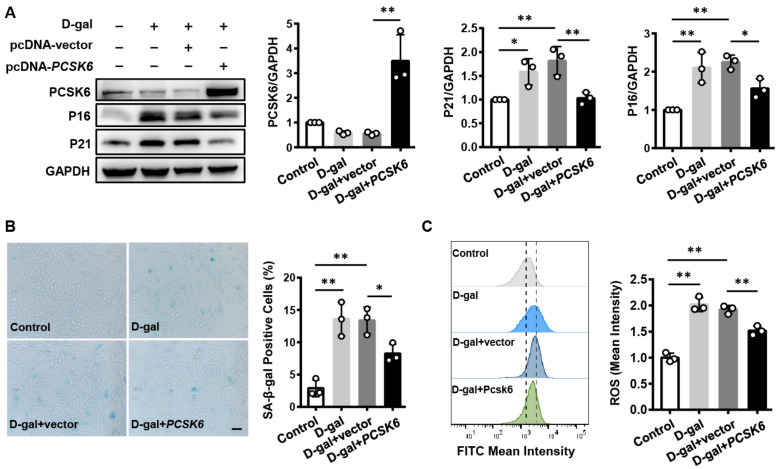
Reverse of cardiomyocyte senescence and dysfunction by *PCSK6* overexpression. (**A**) Western blot analysis of PCSK6, P16 and P21 expression in H9c2 cells treated with D-gal and followed by transfected with a *PCSK6* expression plasmid. Protein levels, normalized to GAPDH, were assessed by densitometry. (**B**) SA-β-gal staining in D-gal-treated H9c2 cells and transfected with a *PCSK6* expression plasmid. Scale bar: 100 µm. Percentages of blue-stained cells were estimated. (**C**) ROS levels in H9c2 cells treated with D-gal and followed by transfected with a *PCSK6* plasmid were analyzed using flow cytometry with the FITC channel. Values are mean ± S.D. * *p* < 0.05, ** *p* < 0.01 vs. Control.

To verify the effect of PCSK6 on cardiomyocyte senescence, we overexpressed *PCSK6* in senescent H9c2 cells induced by D-gal. The cells were treated with D-gal and transfected with a *PCSK6* expression plasmid. We found that P16 and P21 protein expression levels decreased in D-gal-treated H9c2 cells that were transfected with the *PCSK6* expressing plasmid as compared to those in vector-transfected control cells (Figure 5A). The percentage of SA-β-gal positive cells was lower in D-gal-treated cells with *PCSK6* plasmid transfection than in vector-transfected control cells (Figure 5B). Furthermore, ROS levels were also lower in D-gal-treated H9c2 cells with PCSK6 transfection (Figure 5C). These results show that *PCSK6* overexpression rescues senescence and dysfunction induced by D-gal in cultured H9c2 cells.

### 3.6. Identification of the Ddit3 Gene Related to Protein Processing in the Endoplasmic Reticulum (ER) upon Pcsk6-Knockdown

**Figure 6:**
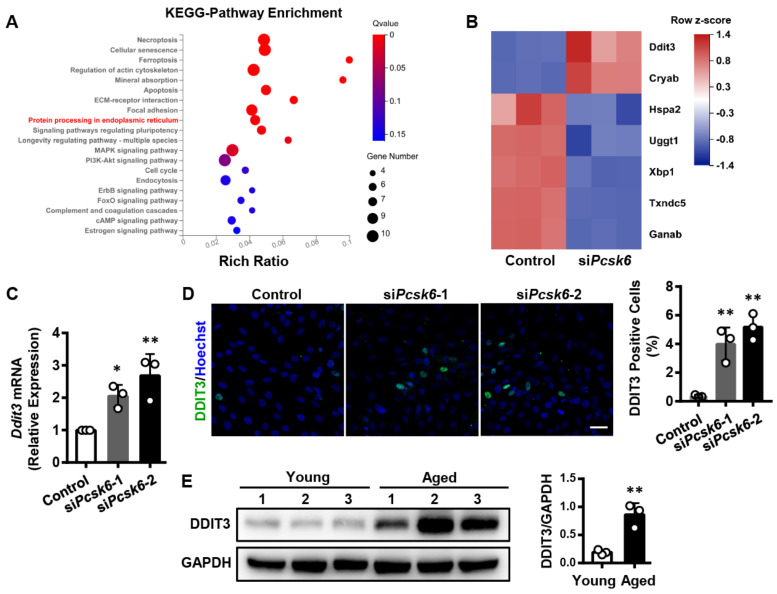
Identification of DEGs by pathway analysis in H9c2 cells with *Pcsk6* knockdown. (**A**) KEGG enrichment analysis of DEGs in signaling pathways. *Y*-axis denotes pathway and *X*-axis denotes rich ratio. The 20 most significantly changed pathways are shown. The targeted pathway in the study is highlighted with red color. The color intensity and size of bubbles indicate Q value and number of genes, respectively. (**B**) A heat map for DEGs in si*Pcsk6*-transfected H9c2 cells. The 7 most significant DEGs in the protein processing in the ER pathway. Red and blue colors represent up- and down-regulated genes, respectively. (**C**) qPCR analysis of *Ddit3* mRNA expression in si*Pcsk6*-transfected H9c2 cells. (**D**) Immunofluorescent staining for DDIT3 (green) and nuclei (blue) in si*Pcsk6*-transfected H9c2 cells. Scale bar: 40 µm. Percentages of DDIT3 positive cells were calculated. Data are mean ± S.D. * *p* < 0.05, ** *p* < 0.01 vs. Control. (**E**) DDIT3 protein levels in hearts from young (3 months, *n* = 3) and aged (24 months, *n* = 3) mice were assessed by densitometric quantification of Western blots. Values are mean ± S.D. * *p* < 0.05, ** *p* < 0.01 vs. Young.

The above results suggested a role of PCSK6 in cardiomyocyte senescence. To understand the underlying molecular mechanisms, we performed RNA sequencing in H9c2 cells transfected with targeting siRNAs, si*Pcsk6.* The data were evaluated by Kyoto Encyclopedia of Genes and Genomes (KEGG) enrichment analysis. We found 20 signaling pathways with most changes. Among them, the protein processing in endoplasmic reticulum was our first target (Figure 6A), since accumulation of misfolded proteins induces ER stress and activates the unfolded protein response (UPR). Under excessive ER stress, the mechanisms triggered by the UPR may not maintain normal ER function and lead to apoptosis. Importantly, ER stress is a primary feature of cardiac aging [28]. We next identified the top 7 differentially expressed genes (DEGs) related to protein processing in the ER (Figure 6B). Among these DEGs, *Ddit3* (also known as CHOP, GADD153, or C/EBP Zeta), considered as an ER stress marker [29], was the most up-regulated gene, suggesting that PCSK6 may be associated with ER stress and protein homeostasis. 

To verify these results, we conducted in vitro and in vivo experiments. qPCR showed that *Ddit3* mRNA levels were elevated in H9c2 cells with si*Pcsk6* treatment (Figure 6C). Immunofluorescent staining detected a higher percentage of DDIT3-positive cells in *Pcsk6*-knockdown cells in comparison to the control cells (Figure 6D). Moreover, Western blot analysis displayed that DDIT3 protein level increased in hearts from aged mice compared to young mice (Figure 6E), indicating a stronger ER stress response in aged hearts. All these data indicate that *Pcsk6* deficiency enhances ER stress in cardiac aging.

### 3.7. PCSK6 Regulates DDIT3 Expression in Tunicamycin (Tm)-Treated Cardiomyocytes

**Figure 7:**
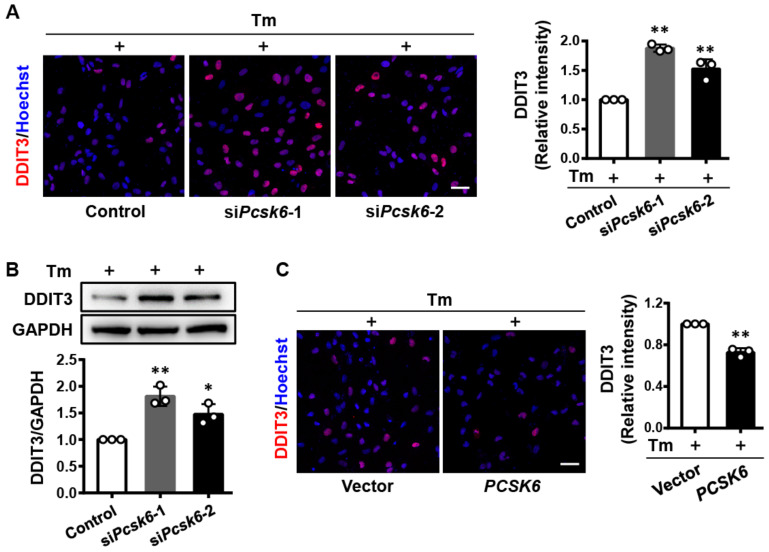
PCSK6 in the regulation of DDIT3 expression in Tunicamycin (Tm)-treated H9c2 cells. (**A**) Immunofluorescent staining of DDIT3 protein (red) and nuclei (blue) in H9c2 cells treated with two sets of si*Pcsk6* and Tm (1 μg/mL). Scale bar: 40 µm. Relative levels were analyzed based on mean fluorescence intensity. (**B**) Western blot analysis of DDIT3 protein expression in *Pcsk6*-knockdown H9c2 cells treated with Tm (1 μg/mL). (**C**) *PCSK6*-overexpressed H9c2 cells treated with Tm (1 μg/mL) were immunostained for DDIT3 (red) and nuclei (blue). Scale bar: 40 µm. Values are mean ± S.D. * *p* < 0.05, ** *p* < 0.01 vs. Control or Vector.

To confirm that PCSK6 regulates ER stress response in cardiomyocytes, we examined DDIT3 expression in *Pcsk6*-deficient or overexpressed H9c2 cells treated with Tm, an ER stress inducer [30]. We transfected H9c2 cells with two sets of si*Pcsk6* and then treated the cells with Tm. By immunofluorescent staining, we found high levels of DDIT3 protein in Tm-treated cells with *Pcsk6* knockdown (Figure 7A). A similar result was shown by Western blotting (Figure 7B). The data indicate that a lack of PCSK6 expression aggravated ER stress induced by Tm. We next overexpressed PCSK6 in Tm-treated H9c2 cells and observed reduced DDIT3 expression levels in immunofluorescent staining (Figure 7C). The results indicate that PCSK6 overexpression inhibits ER stress induced by Tm in H9c2 cells, supporting a role of PCSK6 in regulating DDIT3 expression, thereby mediating ER stress in cardiomyocytes.

## 4. Discussion

In this study, we show that PCSK6 protein expression is reduced in senescent cardiomyocytes in culture and in aged mouse hearts. Knockdown of *Pcsk6* in cardiomyocyte leads to a phenotype of senescence and dysfunction, whereas *PCSK6* overexpression prevents such a phenotype and improves cellular function. Furthermore, *Pcsk6* deficiency increases the DDIT3 level and PCSK6 overexpression suppresses the ER stress in cardiomyocytes. These data indicate that PCSK6 modulates cardiomyocyte senescence through an ER stress response mediated by DDIT3 expression.

Cardiomyocytes are one of the cardiac cell types, comprising >70% of the cardiac tissue mass. Other cell types include endothelial cells, fibroblasts, and blood-derived cells [31,32]. In adult hearts, cardiomyocytes are end-differentiated cells with little capacity for regeneration. In contrast, cardiac endothelial cells and fibroblasts have potential to rejuvenate during adulthood [33]. In this study, therefore, we focused on cardiomyocytes. Unlike replicative cell senescence, cardiomyocytes are more susceptible to stress-induced premature senescence [33]. D-gal as a reducing sugar has been commonly applied for generating the model of aging animal and cellular senescence [34,35,36]. In our study, D-gal-induced cardiomyocyte senescence presents a similar phenotype to replicative senescence, including elevated levels of senescence markers P16 and P21, increased SA-β-gal activity, and up-regulated Il6, as reported previously [13,36,37]. This senescence model provides a suitable experimental setting for studying cardiac aging in vitro.

Cardiomyocyte senescence is generally characterized by DNA damage, mitochondria dysfunction, ER stress, senescence-associated secretory phenotype (SASP), contractile dysfunction, and hypertrophic growth [28]. It is believed that DNA damage is the central cause for cell senescence. Accumulation of ROS may induce cellular DNA damage [38]. We found that *Pcsk6* deficiency increased ROS production, consistent with the feature of cardiomyocyte senescence [28]. ROS levels regulate the cellular signal transduction, gene expression, cell proliferation, senescence, and apoptosis in vitro and in vivo [39]. Increased ROS levels lead to mitochondrial dysfunction in senescent cardiomyocytes and aged cardiac tissues [40]. However, mitochondrial ROS may be considered as a defense mechanism for triggering the adaptive response to promote health and extend lifespan [41]. On the other hand, excessive ROS may induce oxidative damage, leading to the development of aging and age-related diseases [42]. Additionally, SASP is recognized as one of the features of cardiomyocyte senescence. In line with our data, mRNA expression of *Il6*, one of the most important cytokines in SASP, is up-regulated in age-related cardiomyocyte senescence, which is associated with mitochondrial dysfunction, elevated oxidative stress, and apoptosis [43,44]. We show that *Pcsk6* deficiency induces cardiomyocyte senescence and dysfunction. Conversely, *PCSK6* overexpression blunts the senescence phenotype, including increased levels of P16, P21, β-gal activity, and ROS production. These data support an important role of PCSK6 in preventing cardiomyocyte senescence.

In our study, *Pcsk6* knockdown induces cardiomyocyte apoptosis. Previously, a similar finding was reported in cancer cells [23]. The reduction in cardiomyocyte number is a prominent feature in aging rat hearts, which is triggered by mitochondrial-dependent apoptosis [45]. It was reported that age-related cardiac apoptosis may be regulated by microRNA-34a targeting the serine/threonine-protein phosphatase 1 regulatory subunit 10 (*PNUTS*) gene [5]. Apoptosis results in cardiomyocyte loss with increasing age, leading to cardiac dysfunction and aging [4].

Upon cardiac aging, advanced glycation end products (AGEs) accumulate in the cells, which may accelerate the aging process [46]. AGEs are formed by a combination of sugar and protein or lipid, which accrues in the cardiovascular system, causing physiological alterations including myocardial stiffness and calcium signaling, thereby contributing to age-related cardiac dysfunction [47,48]. Our data show that *Pcsk6* knockdown increased cellular AGEs, indicating decreased cardiomyocyte function. AGEs are considered as one of the factors for cell senescence that impair cardiac structure and function.

In our RNA sequencing analysis, the enrichment of DEGs was found in the signaling pathways involving necroptosis, cellular senescence, apoptosis, and protein processing in the ER. Several studies have shown that increased ER stress contributes to cardiovascular disease such as heart failure and atrial fibrillation [49,50,51,52]. In fact, ER stress is one of the hallmarks of cardiomyocyte senescence [28]. Therefore, our study focused on ER-related molecular pathways. We found the most significant increase in Ddit3 in an ER-related pathway [53] in *Pcsk6*-knockdown cardiomyocytes and aged mouse hearts. DDIT3, a C/EBP family member, is a transcriptional factor induced by ER stress [54,55]. Possibly, PCSK6-mediated cardiomyocyte senescence may be caused by an excessive ER stress response. Previous reports showed elevated ER stress in *PCSK6*-deficient prostate cancer cells [23], indicating that PCSK6 has a regulatory effect on ER stress. It has been shown that accumulation of misfolded proteins in the ER enhances DDIT3 expression that induces apoptosis in cardiomyocytes, leading to cardiac dysfunction and heart failure [56,57].

Studies have shown that cells with high levels of DDIT3 undergo apoptosis, whereas a lack of DDIT3 expression prevents cell death associated with stressed ER [29,58]. We observed that cardiomyocyte apoptosis induced by PCSK6 deficiency may be implicated in elevated DDIT3 expression mediated by ER stress [59]. ER is known to be a major site of ROS production [60]. Our data indicate that PCSK6 deficiency increases ROS levels in cardiomyocytes. Possibly, the increased ROS levels caused by PCSK6 deficiency may be associated with ER stress. Further experimental data supported the effect of *PCSK6* on DDIT3 expression in Tm-treated cardiomyocytes [61]. Under ER stress, *Pcsk6* knockdown augments DDIT3 expression, whereas *PCSK6* overexpression suppresses DDIT3 levels, in cardiomyocytes. It is likely, therefore, that reduced PCSK6 levels may increase the susceptibility of ER stress in cardiomyocytes [62,63].

There are several limitations in this study. H9c2 cells were used as a model of cardiomyocytes in culture. These cells may differ from cardiomyocytes in the heart in physiological characteristics. The effect of PCSK6 on cardiac aging uncovered in our study needs to be verified in *Pcsk6*-deficient animal models and in human aging hearts. In addition, PCSK6 is known to play a key role in the TGF-β signaling pathway [18]. It will be important to examine if the role of PCSK6 in cardiac aging involves additional signaling pathways, including mechanisms mediated by TGF-β signaling.

## Data Availability

Not applicable.
